# Linkage and association analysis of nevus density and the region containing the melanoma gene CDKN2A in UK twins

**DOI:** 10.1038/sj.bjc.6600904

**Published:** 2003-06-10

**Authors:** J H Barrett, R Gaut, R Wachsmuth, J A Newton Bishop, D T Bishop

**Affiliations:** 1 Cancer Research UK Clinical Centre, Genetic Epidemiology Division, St James's University Hospital, Leeds, UK

**Keywords:** nevus density, melanoma, CDKN2A, association, linkage analysis

## Abstract

Rare mutations in the CDKN2A gene are highly penetrant for melanoma. Density of nevi is under strong genetic control and high density is a potent risk factor for melanoma. We used linkage and association analysis in adolescent twins from the UK to examine the hypothesis that the region containing the CDKN2A gene also contains a quantitative trait locus influencing normal nevus development. Five markers in the CDKN2A region were genotyped in 115 dizygotic twin pairs, and one marker (D9S942) was genotyped in 103 monozygotic twin pairs, all of whom had been phenotyped for nevus density. Linkage analysis showed no evidence of a quantitative trait locus influencing nevus density in this chromosomal region. A model partitioning the variation in phenotype into within- and between-twin pair components showed weak evidence of association between higher nevus density and longer mean length of the two D9S942 alleles (*P*=0.01). This relation, which was also observed in an earlier Australian twin study, could be because of the linkage disequilibrium between D9S942 and a neighbouring functional locus. Further investigation of this region is warranted in large-scale linkage or association studies.

Rare mutations in the CDKN2A gene at chromosome 9p21 underlie disease susceptibility in up to 40% of multiply affected melanoma families (Harland *et al*, 1997). High density of common melanocytic nevi is also a potent risk factor for melanoma in the general population, and nevus density is under strong genetic control. A natural question is therefore whether the CDKN2A gene also influences the development of normal nevi. In a recent study of 12-year-old Australian twins, Zhu *et al* (1999) addressed this question by investigating linkage of a putative quantitative trait locus (QTL) for mole density to the polymorphic (CA)_*n*_ repeat marker D9S942 within CDKN2A. They found some evidence for linkage. Correlations in log mole counts between dizygotic (DZ) twins sharing 0, 1 and 2 alleles at this locus identical by descent (IBD) were estimated as 0.44, 0.64 and 0.74. As would be expected if a QTL for mole count exists close to this marker, the correlations were heterogeneous (*P*=0.002, based on 199 twin pairs), increasing as the number of alleles shared IBD increased. The authors went on to fit a path model to further investigate this locus, and they estimated that the QTL accounted for 27% of the variance in log-transformed mole count. Phenotype was also regressed on the mean length (in base pairs) of the two D9S942 alleles. Results were described for total flat moles, and although this explained less than 1% of the variation, there was significant evidence of association (*P*=0.004).

Following on from this, we have conducted a study of similar design in the UK (Wachsmuth *et al*, 2001). Comparisons between the two sets of twins in the UK and in Australia, with similar ancestry but very different exposures to ultraviolet light, have allowed us to investigate the relative contributions of genes and environment (in particular sun exposure) to nevus development. It was found that Australian children had on average approximately 40% more moles than UK children, presumably reflecting the greater exposure to the sun. Estimates of heritability from the two studies were however remarkably similar; in the UK 65% of the variance in nevus density was estimated to be attributable to additive genetic effects compared with 68% in Australia (Wachsmuth *et al*, 2001).

Here we report on the results of a linkage and association analysis in the UK twins.

## MATERIALS AND METHODS

The twin study is described in detail elsewhere (Wachsmuth *et al*, 2001). Ethical committee approval was obtained for the study from a multiregional ethics committee and all local ethics committees. Briefly, 221 twin pairs between the ages of 11 and 18 years were recruited via schools in two regions of England. The twins' moles were counted according to size by a dermatologist (RW), and the children and their parents were asked to supply a blood sample for DNA testing. To facilitate comparisons with the Australian study, the counting method was assessed directly against that of the key Australian research nurse, who also counted the nevi in the first 16 twin pairs of our sample. Counts were converted to nevus density by dividing by estimated body surface area (a function of height and weight).

**Figure 1:**
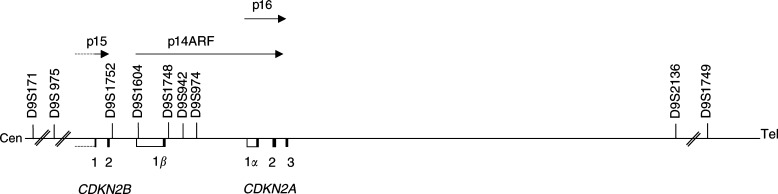
Genetic map of the *CDKN2* locus. The positions of several 9p microsatellite markers are shown. The exons of *CDKN2B*, *CDKN2A* and exon *1β* are denoted by black rectangles, with the primary transcripts indicated by arrows. The promoters of each gene are shown with white rectangles; the promoter of p15 is illustrated as a partial rectangle (dotted line) because the size of the promoter is not known.

Five markers (D9S171, D9S1748, D9S942, D9S974 and D9S1749) spanning a 5.7 Mb region round CDKN2A were genotyped in the 115 DZ twins. The three central markers are in linkage disequilibrium and span a region of less than 7 kb within the CDKN2A gene (see Figure 1

). For each twin pair, it was determined whether they shared 0, 1 or 2 haplotypes IBD. Where it was necessary to resolve ambiguities in IBD sharing, or inconsistencies because of genotyping error or possible recombination, parents were also typed. Marker D9S942 was also genotyped in the monozygotic (MZ) twin pairs.

All statistical analyses, including the linkage analysis described below, were carried out in Stata Statistical Software release 7.0 (Stata Corporation, 2001). Nevus counts and densities were log transformed to produce a less skewed phenotype distribution. They were regressed on age and sex, and residuals from the regression were used in further analyses. Intraclass correlation coefficients were then calculated for each of the three haplotype-sharing groups among the DZ twins and for the MZ twins. The Haseman–Elston method, regressing the squared difference in phenotype on the number of haplotypes shared IBD, was applied to test for linkage (Haseman and Elston, 1972) using the DZ twins.

To confirm or refute the findings of Zhu *et al* (1999), the association of an individual's phenotype with the mean length of their two D9S942 alleles was also examined. This was done by modelling phenotype as a linear function of mean allele length (with a fixed covariate) and including the twin pair as a random effect. The model can be written as

*y*_*ij*_ = *α*+*γ*_*i*_+*βx*_*ij*_+*ɛ*_*ij*_

where *y*_*ij*_ is the phenotype for twin *j* from pair *i*, *α* is the overall mean, γ_*i*_ is a random effect term for twin pair *i* and is assumed to be normally distributed with mean 0 and variance *σ*_u_^2^, *x*_*ij*_ is the mean allele length for twin *j* from pair *i* and *ɛ*_*ij*_ is the individual level error assumed normally distributed with mean 0 and variance *σ*_e_^2^. This is the simplest form of multilevel model, as described by Goldstein (1995). The level 1 residuals (*ɛ*_*ij*_) are assumed to be independent between observations and of the other terms in the model, and the level 2 residuals (*γ*_*i*_) are assumed to be independent across twin pairs. The parameters *β*, *σ*_u_^2^ and *σ*_e_^2^ were estimated here by maximum likelihood (*xtreg* with the *mle* option in Stata). Under the null hypothesis of no association, *β*=0. The analysis was performed using DZ twin pairs only and then using all twin pairs, since the assumptions of a single distribution for the level 1 residuals may be violated by grouping together the MZ and DZ twins.

## RESULTS

Descriptive statistics are presented in Table 1

**Table 1:** Descriptive statistics of the nevus phenotypes

	**Number of observations**	**Mean**	**s.d.**	**Skewness**	**Median**	**Lower and upper quartile**
Total number of nevi^a^	426	90.7	59.2	1.66	77	50–118
Density of nevi^a^	426	70.4	43.5	1.56	70	39–88
Density of nevi 2–5 mm	442	30.9	24.4	1.83	24	14–39
Number of atypical nevi	442	1.00	1.68	3.34	0	0–1

a Nevi less than 2 mm wide were not measured on eight twin pairs.

for the various nevus phenotypes. It can be seen that the distributions were positively skewed, but after log transformation, the total count and densities were approximately normally distributed.

**Figure 2:**
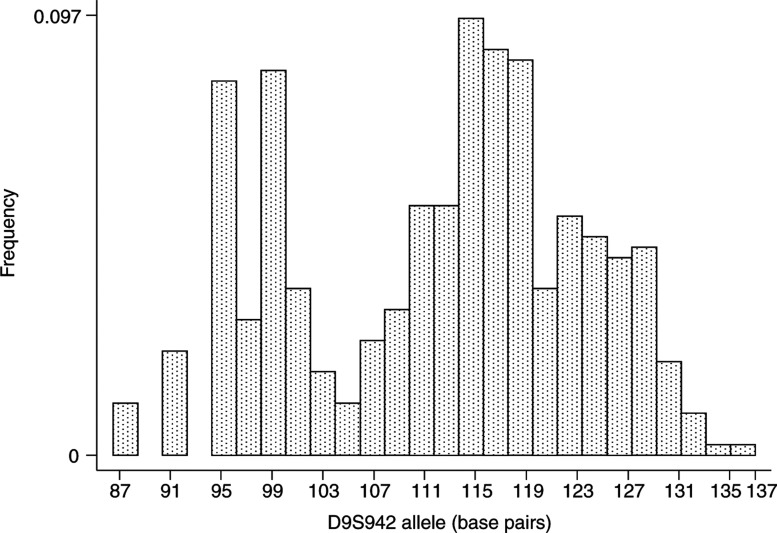
Frequency of the D9S942 alleles in 218 twins (one twin from each pair).

Overall for these five markers genotyping was 99% complete on the 115 DZ twin pairs, and a total of 162 parents were also typed. For D9S942, genotyping was complete for 103 (97%) twin pairs. Allele frequencies for D9S942 are shown in Figure 2

and were very similar to those reported by Zhu *et al* (1999). In 74 DZ families, the number of haplotypes which shared IBD was clear from consistent sharing of alleles identical by state between the twins, and in the remaining families parental data were used. For two twin pairs, DNA quality was poor and genotyping was not sufficiently complete to assign IBD sharing. Five recombination events were detected between markers D9S171 and D9S1748 (separated by 5.5 Mb), and IBD sharing at the remaining loci is reported here.

Among the DZ twins, 32 (28%) twin pairs shared 0, 59 (52%) shared 1 and 22 (19%) shared 2 haplotypes IBD, which was consistent with expected proportions (1 : 2 : 1) under Mendelian segregation. Correlations in nevus density between co-twins are shown in Table 2

**Table 2:** Correlations in mole counts between twin pairs and results of Haseman–Elston analysis

	**Correlations between twins in pair (95% CI^a^)**					
	**0 haplotypes shared (*n*=32)**	**1 haplotype shared (*n*=59)**	**2 haplotypes shared (*n*=22)**	**DZ twin pairs (*n*=115)**	**MZ twin pairs (*n*=106)**	**HE regression coefficient^b^ (95% CI)**
Total number^c^ of nevi	0.73	0.62	0.36	0.63	0.94	0.04
	(0.57, 0.89)	(0.46, 0.78)	(0, 0.75)	(0.51, 0.74)	(0.92, 0.96)	(−0.08, 0.16)
Density of nevi^c^	0.73	0.60	0.23	0.61	0.94	0.04
	(0.57, 0.89)	(0.43, 0.77)	(0, 0.65)	(0.49, 0.72)	(0.91, 0.96)	(−0.08, 0.15)
Density of nevi 2–5 mm	0.65	0.55	0.27	0.53	0.91	0.07
	(0.44, 0.85)	(0.37, 0.73)	(0, 0.66)	(0.40, 0.66)	(0.88, 0.94)	(−0.15, 0.30)
Number of atypical nevi	0.39	0.44	0	0.32	0.51	0.14
	(0.10, 0.69)	(0.23, 0.65)	(0, 0.42)	(0.16, 0.49)	(0.37, 0.65)	(−0.05, 0.33)

a CI=confidence interval.

b Haseman–Elston regression coefficient: this is the increase in squared difference in mole count per number of haplotypes shared (0, 1 or 2).

c Nevi less than 2 mm wide were not measured on some twin pairs; these analyses are based on 110 DZ pairs (32, 56, 20 sharing 0, 1 and 2 haplotypes and two unknown) and 103 MZ pairs.

, which includes for comparison the corresponding estimates for MZ twins. For moles between 2 and 5 mm in diameter, the correlation in nevus density in DZ twins was 0.53 (95% confidence interval 0.40–0.66). The estimated correlation coefficients for the 0, 1 and 2 haplotype sharers were 0.65, 0.55 and 0.27, respectively. Thus, there was no greater correlation in those sharing more haplotypes at this locus and hence no support for the existence of a QTL in this region from this analysis. Lower estimated correlations in those sharing more haplotypes were also found for the other measures considered (total nevus count, total nevus density and number of atypical nevi). The Haseman–Elston regression coefficients, which would be negative in the presence of linkage, are also shown in the table along with their confidence intervals.

Based on DZ twins, the random effect model showed weak evidence of association between nevus density and mean D9S942 allele length (regression coefficient *β* estimated to be 0.012, 95% confidence interval (0.000, 0.023), *P*=0.04). Note that this is a regression coefficient relating phenotype to the individual twin's mean allele length having allowed for the (random) effect of the twin pair and is based on both the within- and between-twin pair information. Longer allele lengths were thus associated with greater nevus density. In keeping with the findings in the Australian study, this mean allele length only accounted for 1% of the overall variation in phenotype. It explained 4% of the within-twin pair variation and less than 1% of the between-pair variation.

Including MZ twin pairs in the analysis, the estimated coefficient is 0.013 (0.004, 0.022; *P*=0.01). Since there is no variation in mean allele length between the two MZ twins in a pair, the MZ pairs contribute no within-pair information but do contribute to the estimate of *β*.

Figure 3

**Figure 3:**
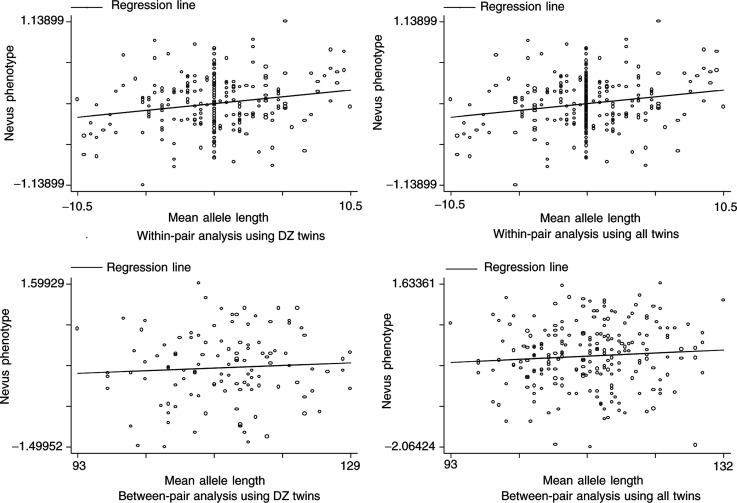
Scatterplots and regression lines showing the relation between nevus phenotype and mean D9S942 allele length both within and between-twin pairs. The phenotype is the residual from regression of the log-transformed density of nevi (2–5 mm in diameter) on age and sex. The top two graphs plot the difference between the individual twin's phenotype and that of the twin pair against the difference in the twin's mean allele length and that of the twin pair; the bottom graphs plot for each twin pair their mean phenotype against mean allele length.

shows

- the difference between the individual twin's phenotype and the mean for the twin pair plotted against the difference between the individual twin's mean allele length and the mean for the twin pair in DZ twins (a within-pair analysis).
- As (a), but for all twin pairs.
- The mean phenotype for a twin pair plotted against the mean allele length for the twin pair in DZ twins (a between-pair analysis).
- As (c) but for all twin pairs.

## DISCUSSION

The linkage analysis in this study offers no support for the existence of a QTL influencing nevus density in this region of chromosome 9. The observed pattern of correlations in twins sharing 0, 1 and 2 haplotypes IBD is in fact opposite to that expected in the presence of such a locus, and our data are only consistent with a very modest negative slope in the Haseman–Elston regression. In contrast our association analysis provides some support for a relation between mean D9S942 allele length and nevus density, and the fact that this relation is found within twin pairs indicates that the result cannot be attributed to population stratification.

The discrepancy between our linkage results and those of Zhu *et al* (1999) could be because of lack of power, since the current sample is not large enough to rule out the existence of a QTL of modest effect. To achieve 80% power to detect a QTL accounting for 30% of the variability in phenotype approximately 800 sibling pairs would be required (a substantially larger study than any conducted to date), assuming no recombination between the QTL and the observed markers and using a significance level of 0.05 (Amos and Elston, 1989). Although there could be differences between Australian and UK children in the influence of genes at 9p21 on nevus density, this seems unlikely in view of their common ancestry and the similarity in heritability estimates from the two populations.

Alternatively, the finding of linkage in the sample of Australian twins and the weak evidence for association in both studies could be false-positive results. The evidence for association, although present in both studies, is not altogether convincing. In both cases only a modest effect on phenotype was found, and Zhu and colleagues estimated that this accounted for less than 6% of the variation in phenotype attributable to the linked QTL. We have done some *posthoc* power calculations on the association analysis using the methods and program of Dupont and Plummer (1998). These calculations are only approximate as they ignore the correlated nature of the data and treat the problem as one of simple linear regression. We calculate that our study using all twins has approximately 79% power to detect an effect of the size found (slope of 0.012), although using only the DZ twins, the power is reduced to 51%.

A further consideration is that an association with mean number of CA repeats is surprising, since the length of such markers is unlikely to affect gene function. However, D9S942 is so polymorphic that some sort of structure or grouping of alleles must be imposed before association can be investigated. No detailed justification was presented by Zhu *et al* for their choice of analyses, and the analyses they present are all based on regression of phenotype on allele length in one form or another. We found, like Zhu *et al*, that alternative models regressing phenotype on the length of the shorter allele, the longer allele or both alleles fit the data less well than using mean length. It is possible that the observed association is because of linkage disequilibrium between D9S942 and another functional locus. No association was found between nevus density and the other four markers studied.

The age matching inherent in a twin study is advantageous, since nevus development is age-dependent, but it is difficult to achieve very large sample sizes using twins. This region clearly warrants further investigation in much larger association and linkage studies using alternative study designs.
